# The English System of Diet for Nervous Exhaustion

**Published:** 1887-02

**Authors:** 


### THE ENGLISH SYSTEM OF DIET FOR NERVOUS EXHAUSTION.

Americans, during the ten minutes they allow themselves for breakfast and lunch, and the twenty minutes for dinner, eat, as a rule, "the best of everything."

This rule, doubtless a very good one to live by when in robust health, fails them in time of sickness. When one feels ill he will be reminded of a very old proverb, which with a slight modification may read: "The well man's meat is the sick man's poison."

In nearly every case that comes into the physician's hands there is required a change of diet. The chances are that the origin of the trouble is to be sought in the living and in the occupation. Then a change of occupation and a change of diet are recommended along with the proper medication.

If the living of the patient has been too concentrated and he is suffering from plethora, biliousness and threatened gout, he may be put upon the Vegetarian regime. So, when there is obstinate constipation, sometimes a return to first principles in the shape of coarse bread and fruit may be beneficial.

Again, when the body is wasted by fever or in phthisis and the digestive organs are weak, there is a return to nature by another path—the Milk diet and soluble food. One begins life over again. We become as little children.

For diabetes and for the reduction of corpulence the diet of the patient is restricted to meat and salads, or if the case indicate insufficient aliment and poor blood, the rare beef steaks and the juicy mutton-chops seem equal to the gifts of the gods. When the digestion is feeble, peptones and peptonoids take their place

There is at present in America, and, indeed, all over the World, a large class of people who are ailing and who neither the vegetarian diet, nor the milk diet, nor the beef diet can cure. Neither are they made happy by the well man's diet, "good living," "the best of everything." These are the brain-workers, the nervous, neurasthenics—all, in fact, who are suffering from exhausted nerves. For a number of years past, from the whole medical profession, the question has gone up: "What is the best brain and nerve food?" The answer has come, at last, from England, and appears in Chapter XXVIII of a new "Manual of Dietetics," entitled "Food in Neurosal Affections." The chapter is short, and the substance of it may be brought down still shorter and comes to this: Stop eating lean meat, and live on fish and bread and butter. "Experience," the author says, "has taught us the fact, even before physiological chemistry could tell us why, that fat and fish are the foods which are especially indicated. A phosphoric fat has to be supplied to the nervous system."

It is an old fallacy, of course, that the living exclusively on fish is conducive to intellectuality; else, say those who vanquish Berkeley with a grin, the South Sea Islanders and the fishermen of our coasts would be philosophers. But although fish-eating cannot make philosphers either of seals or fishermen, it does not follow that this phosphorescent meat is not useful to recuperate exhausted nerve.

The main feature, however, is not the fish but the fat. Lecithin, that conspicuous component of brain and nerve—a substance unknown to the vegetable world—is fat. Butter, cream, and cod-liver oil, and milk, fat bacon, and the yolk of eggs should constitute the chief factor in the combination diet. Fish furnishes the phosphorous and it also furnishes sufficient nitrogen for suppoit.

"When we consider," he says further, "that the pabulum of the nervous system is a phosphorised fat we can comprehend why the plan of treating cases of cerebral exhaustion by liberal quantities of lean meat has turned out a failure. Albuminoids do not supply the required material for the intended purpose, while in their metabolism they furnish matters which may be called hepatic mal-products or liver stuffs which possess very irritating and toxic properties as regards the brain, consequently a highly nitrogenised dietary is not only without advantages but actually posseses positive drawbacks. The brain is not fed thereby but, in its week condition, is annoyed and vexed by these liver stuffs."

An American patient is cited who lost his bilious headache by striking out of his diet the flesh of all animals except fish. "He lives on a milk and farinaceous dietary with butter plus the fish."

Thus this nervous American keeps perpetual Lent and thrives, while any momentary backsliding toward the flesh-pots brings its own punishment. The Catholics are right, and Lenten fare has its uses. Let us not wait for Lent, but try it at once—on our patients.

In the meantime, however, we cannot forget the physicians main reliance, thus far, in nearly all cases of weakness from whatever cause has been upon the albumenoids.—*Jour. of Reconstructives.*

